# Association between Dietary Quality and Prediabetes based on the Diet Balance Index

**DOI:** 10.1038/s41598-020-60153-9

**Published:** 2020-02-21

**Authors:** Dingliu He, Yanan Qiao, Suting Xiong, Siyuan Liu, Chaofu Ke, Yueping Shen

**Affiliations:** 1 Department of Epidemiology and Biostatistics, School of Public Heath, Medical College of Soochow University, 199 Renai Road, Suzhou, 215123 P.R. China; 2 Department of Clinical Nutrition, Yancheng No.1 People’s Hospital, Yancheng, 224001 China

**Keywords:** Nutrition disorders, Nutrition

## Abstract

Dietary quality is an important factor influencing prediabetes, but few studies have applied the Chinese Diet Balance Index (DBI-16) to evaluate the dietary quality of individuals with prediabetes and explore the associations between dietary quality and prediabetes. In our study, the lower-bound score, higher-bound score and diet quality distance, were respectively calculated to assess dietary quality based on each food group. Logistic regression was used to calculate the odds ratio (OR) and 95% confidence interval (95%CI) of unfavorable dietary quality leading to prediabetes in every subgroup. The results were shown that individuals with prediabetes had excessive intake in the categories of cereals, salt and inadequate intake in vegetables, fish and diet variety than participants without prediabetes (all P < 0.01). Unfavourable dietary quality was significantly associated with an increased risk of prediabetes (OR: 1.45, 95%CI: 1.29–1.63), especially among the subjects who lived in rural areas (OR: 1.63, 95%CI: 1.25–1.76), those who had abdominal obesity (OR: 1.58, 95%CI: 1.36–1.85), those who smoked (OR: 1.58, 95%CI: 1.30–1.93), those who consumed alcohol (OR: 1.57, 95%CI: 1.28–1.93) and those who did not drink tea (OR: 1.64, 95%CI: 1.42–1.88). In Conclusion, unfavourable dietary quality was significantly associated with an increased risk of prediabetes.

## Introduction

Prediabetes and diabetes have become a serious global problem in recent decades, along with the development of the social economy and changes in people’s lifestyles. Prediabetes is characterised by glucose levels that do not meet the criteria for diabetes but that are too high to be considered normal^1^. The International Diabetes Federation (IDF) reported that there were approximately 350 million people with prediabetes worldwide and 48.6 million people with prediabetes in China^2^. People with prediabetes have a high risk of developing diabetes, and 70~90% of individuals with prediabetes will eventually develop diabetes^3^.

Dietary quality is an important factor influencing prediabetes and diabetes. Healthier dietary intake can reduce the risk of the progression from prediabetes to diabetes by 40~70%^3^. Among people with prediabetes who changed their dietary model, 40.5% achieved normal glucose tolerance, and their fasting plasma glucose level, body mass index (BMI), glycated haemoglobin (HbA1C) level and blood pressure decreased significantly as well^4^. Therefore, several dietary evaluation indexes have been developed around the world in recent years. In China, researchers mainly use the Diet Balance Index-16 (DBI-16) to assess human dietary quality, and the index was updated based on the latest Chinese Dietary Guidelines and the Chinese Balanced Diet Pagoda^5^. The DBI-16 can be used to evaluate the overall dietary quality mainly through three indicators: the lower-bound score (LBS), higher-bound score (HBS), and diet quality distance (DQD). The LBS represents insufficient intake, HBS represents excessive intake and DQD represents the overall imbalance of the diet^5^.

Traditional dietary evaluation is based on a single nutrient or food index, which cannot accurately reflect the complexity of the diet. It is also difficult to evaluate dietary quality as a whole. The advantage of the DBI-16 is that it can reflect not only insufficient dietary intake but also excessive dietary intake. Moreover, the DBI-16 can evaluate the quality of the diet as a whole. In addition, previous researchers have paid little attention to the relationship between overall dietary quality and prediabetes, instead focusing on the effect of a single nutrient on prediabetes^6,7^. To better explain the relationship between overall dietary quality and prediabetes, we explored the association between dietary quality and prediabetes by using the DBI-16 to evaluate dietary quality.

## Materials and Methods

### Study design and population

The data we used were from the China Health and Nutrition Survey (CHNS), an ongoing open cohort study. The surveys were started in 1989, and the population has been followed up every two or four years using a multistage random clustering method to obtain a sample of approximately 7200 households including over 30000 individuals in 15 provinces. As a longitudinal study, the CHNS aims to identify changes in geography, public resources, economic development and health indicators. Food markets, health facilities and other social services are also assessed in the CHNS^8^. The data we used were obtained in 2009. After excluding participants with missing dietary data or HbA1C data, HbA1C > 6.4%, diagnosed or self-reported diabetes, or implausibly low or high energy intake levels (<800 or >4000 kcal), 7693 subjects aged 18 years or older were included in the final analysis.

### Dietary data collection and food groups

The dietary intake of the subjects was recorded through the 3-d 24-h recall method at the individual level, and a food inventory was recorded at the household level over the same three-day period^9^. For the 24-h recall, a trained interviewer recorded the consumption of all food in a face-to-face interview, combining subjects’ self-reported values with the total household consumption. We categorized each food consumed into groups according to the associated food code and nutrient profile^5,10^. Because there was insufficient data on sugar used as a condiment, sugar was not included in our study.

### Diet Balance Index-16

The purpose of the Chinese DBI-16 is to enable the assessment of overall dietary quality in the Chinese population. The DBI-16 was modified from the DBI-07, and the DBI-16 has more specific energy assignment levels that can comprehensively reflect the dietary quality of the population; however, there are no differences in the main techniques and methods of evaluating the dietary quality of people. The DBI-16 has seven components from the most recent Chinese Dietary Guideline and the Chinese Food Pagoda, namely (range of values): (1) cereals (−12-12); (2) vegetables (−6-0); fruits (−6-0); (3) milk and dairy products (−6-0), soybean and soybean products (−6-0); (4) animal foods (−4-4 for meat, -4-0 for fish, -4-4 for eggs); (5) empty energy food (0-6 for oil, 0-6 for alcohol); (6) condiments (0-6); and (7) diet variety (−12-0)^5^. A score of 0 is given when the food intake meets the recommendation of the dietary guidelines. The negative or positive scores indicate that recommended level is not met or is exceeded, respectively. The DBI-16 is further divided into 12 food subgroups, which are used to calculate the score for diet variety.

After calculating each DBI-16 score, we further calculated three indicators of dietary quality. The LBS was defined as the absolute value of the sum of all negative scores, indicating insufficient intake. The HBS was defined as the sum of all positive scores, indicating excessive intake. The DQD was defined as the sum of the LBS and HBS and evaluated whether the individual’s food intake was balanced. In our study, the ranges of the HBS, LBS, and DQD were 0–38, 0–60, and 0–78, respectively. A score of 0 indicates excellent dietary intake (no problems), a score that is less than 20% of the total score indicates good dietary intake (almost no problems), a score that is 20–40% of the total score indicates acceptable dietary intake (low level of problems), a score that is 40–60% of the total score indicates poor dietary intake (moderate level of problems), and a score greater than 60% of the total score indicates the worst dietary intake (high level of problems)^11^, which is also defined as an unfavourable dietary quality.

### Other variables

The blood pressure value was the mean of three measurements. People were diagnosed with hypertension when their systolic pressure was ≥140 mmHg or their diastolic pressure was ≥90 mmHg^12^, and they were divided into two groups (Yes/No). Smoking status was also divided into two groups (Yes/No). Age was divided into three groups, namely, young people aged 18–44 years old, middle-aged people aged 45–59 years old and elderly people aged 60 years old and older. BMI was divided into four levels based on the Chinese Working Group on Obesity^13^: underweight (kg/m^2^): BMI < 18.5; normal: BMI 18.5–23.9; overweight: BMI 24.0–27.9; and obesity: BMI ≥ 28.0. Waist circumference (WC) and HbA1C were divided into two groups, and WC ≥ 85 cm for women or WC ≥ 90 cm for men was used to diagnose abdominal obesity^14^. Activity level was estimated based on subjects’ self-reported activities and duration (occupational, transportation, and physical exercise), and they were divided into six levels: very light, light, moderate, heavy, very heavy and unable to be active. When 6.4% ≥ HbA1C ≥ 5.7%, people were diagnosed with prediabetes, and HbA1C < 5.7% indicated the absence of prediabetes^1^.

### Statistical analysis

The statistical data analysis software package SAS 9.4 (SAS Institute Inc., Cary, North Carolina, USA) was used for the data analyses. Means and standard deviations (SDs) are used to describe the normally distributed continuous variables (e.g., age, BMI, WC and DBI scores for each component). We used a t test to detect the differences in the DBI score for each component between subjects with and without prediabetes. A chi-square test was used to detect the differences in the rates of sex, location, education, hypertension, smoking, drinking tea and and drinking alcohol. After adjusting for sex, age, location, abdominal obesity, BMI, education, hypertension, smoking, alcohol consumption, drinking tea, energy and activity, multivariable logistic regression was used to calculate the odds ratio (OR) and 95% confidence interval (95%CI) of unfavorable dietary quality leading to prediabetes in every subgroup. The significance level was set at 0.05 (two sided).

## Results

### Characteristics of the participants

The characteristics of the participants are presented in the two cohorts stratified by prediabetes status in Table 1. There were 7693 participants in this study, and the overall prevalence of prediabetes was 33.0%. Significant differences were found between the two groups in the HBS, LBS and DQD (P < 0.01). Compared with the group without prediabetes, the group with prediabetes had a higher HBS, LBS and DQD; were older; and had higher proportions of individuals who smoked and drank tea. In addition, participants with prediabetes were more likely than people without prediabetes to live in rural areas and have hypertension, were less likely to be highly educated, and had higher BMIs and WCs.

**Table 1:** Characteristics of the study participants stratified by prediabetes status.

Characteristics	Prediabetes	No Prediabetes	*P* value
Subjects (n, %)	2541 (33.0)	5152 (67.0)	
Female (n, %)	1348 (53.0)	2788 (54.1)	0.3783
Age (years)	54.6 ± 14.1	47.0 ± 14.9	<0.0001
Urban (n, %)	766 (30.1)	1708 (33.2)	0.0079
Education (n, %)	545 (21.5)	1283 (24.9)	0.0008
Hypertension (n, %)	917 (36.1)	1086 (21.1)	<0.0001
Smoked (n, %)	834 (32.8)	1530 (29.7)	0.0052
Alcohol (n, %)	823 (32.4)	1674 (32.5)	0.927
Tea (n, %)	926 (36.4)	1754 (34.1)	0.0379
BMI (kg m^−2^)	24.0 ± 3.5	22.7 ± 3.2	<0.0001
WC (cm)	85.1 ± 10.0	80.5 ± 9.8	<0.0001
HBS	13.9 ± 5.2	13.5 ± 5.3	0.0010
LBS	26.7 ± 7.1	25.8 ± 6.6	<0.0001
DQD	40.6 ± 9.4	39.3 ± 8.6	<0.0001

### Scores for the DBI-16 components and the percentages of participants with each score in the two groups

Table 2 shows the scores for the DBI-16 components and the percentages of participants with each score. Excessive intake was common in both groups in the categories of cereals, meat, oil and salt, with 86.8%, 45.1%, 61.7%, and 63.5% of people with prediabetes having positive scores, respectively. Moreover, 87.2%, 56.4%, 62.5%, and 59.6% of the people without prediabetes also had positive scores, respectively. In contrast, inadequate intake of vegetables and fruits, intake of dairy, diet variety, intake of fish and intake of eggs were also common, with 65–99% of the people in the two groups having negative scores. The intake of soybeans increased, and the intake level of 41.7% of the people with prediabetes and 40.4% of the people without prediabetes did not meet the recommendation. People without prediabetes accounted for lower percentages of the maximum scores for excessive intake and inadequate intake (except for dairy and eggs).

**Table 2:** Scores for the DBI-16 components and the percentages of participants with each score in the two groups.

Components	Score range	Groups	Score												
			(−12)-(−11)	(−10)-(−9)	(−8)-(−7)	(−6)-(−5)	(−4)-(−3)	(−2)-(−1)	0	(1)-(2)	(3)-(4)	(5)-(6)	(7)-(8)	(9)-(10)	(11)-(12)
Cereals	(−12)-(12)	No Prediabetes	0.06	0.10	0.14	0.47	1.22	3.65	7.2	11.26	13.59	13.28	10.17	7.38	31.51
		Prediabetes	0.08	0.04	0.31	0.83	1.69	3.54	6.73	9.25	10.78	11.96	11.73	8.03	35.03
Vegetables	(−6)-(0)	No Prediabetes				2.08	27.26	42.47	28.20						
		Prediabetes				3.39	30.42	39.83	26.37						
Fruits	(−6)-(0)	No Prediabetes				71.25	14.40	7.34	7.01						
		Prediabetes				72.29	13.89	7.95	5.86						
Dairy	(−6)-(0)	No Prediabetes				93.97	3.84	1.80	0.39						
		Prediabetes				91.19	5.15	3.39	0.28						
Soybean	(−6)-(0)	No Prediabetes				36.70	1.17	2.50	59.63						
		Prediabetes				38.49	1.10	2.13	58.28						
Meat	(−4)-(4)	No Prediabetes					17.12	12.97	13.47	14.65	41.79				
		Prediabetes					25.77	15.11	14.05	12.67	32.39				
Fish	(−4)-(0)	No Prediabetes					52.66	20.42	26.92						
		Prediabetes					58.17	19.64	22.2						
Egg	(−4)-(4)	No Prediabetes					39.83	27.08	11.34	11.80	9.95				
		Prediabetes					36.97	26.84	10.67	13.77	11.72				
Oil	(0)-(4)	No Prediabetes							37.48	20.69	16.48	25.35			
		Prediabetes							38.29	19.79	15.47	26.44			
Salt	(0)-(4)	No Prediabetes							40.39	34.22	15.43	9.96			
		Prediabetes							36.48	31.25	18.97	13.30			
Alcohol	(0)-(4)	No Prediabetes							91.93	1.96	0.85	5.26			
		Prediabetes							91.22	2.24	0.62	5.90			
Diet variety	(−12)-(0)	No Prediabetes	0.02	4.76	32.57	43.51	17.10	1.98	0.06						
		Prediabetes		7.48	34.98	39.20	15.59	2.67	0.08						

### Distribution of DBI-16 indicators among the participants

Table 3 presents the distribution of DBI-16 indicators among the participants. The LBS score indicates inadequate intake levels, with 61.51% of people with prediabetes having a moderate or high prevalence of inadequate food intake. This figure was 58.52% in people without prediabetes. The distribution of HBS indicates that 36.99% of the people with prediabetes had a high level of excessive food intake, while 35.25% of the people without prediabetes had a high level of excessive food intake. According to the distribution of the DQD, an indicator used to evaluate the overall imbalance in dietary intake levels, more than 83.2% of people with prediabetes had moderate or high levels of imbalanced food intake. Moreover, the findings indicated that 81.4% of people without prediabetes had moderate or high levels of imbalanced food intake.

**Table 3:** Distribution of DBI-16 indicators among the participants.

	Indicator	Range		Mean ± SD	Distribution of diet quality (%)				
					No problem	Almost no problem	Low level	Moderate level	High level
Inadequate intake	LBS	0~60	Prediabetes	26.7 ± 7.1	0	1.93	36.56	52.3	9.21
			No Prediabetes	25.8 ± 6.6	0	1.3	40.18	51.44	7.08
Excessive intake	HBS	0~38	Prediabetes	13.9 ± 5.2	0.35	10.51	52.15	31.72	5.27
			No Prediabetes	13.5 ± 5.3	0.43	12.26	52.06	30.18	5.07
Overall unbalance	DQD	0~78	Prediabetes	40.6 ± 9.4	0	0.35	16.45	55.34	27.86
			No Prediabetes	39.3 ± 8.6	0	0.19	18.42	60.87	20.52

### Comparison of DBI-16 scores (mean ± SD) with components between the two groups

Table 4 shows the DBI-16 score for every component. People with prediabetes had more excessive food intake in the cereal and salt categories and had more inadequacy in the vegetables, fish and diet variety categories than participants without prediabetes. In contrast, people with prediabetes had a slightly more reasonable intake of dairy, meat and eggs.

**Table 4:** The comparison of component DBI-16 scores between the groups.

	No prediabetes	Prediabetes	*P* value
Cereals	6.72 ± 4.72	7.05 ± 4.87	0.0004
Vegetables	−1.67 ± 1.39	−1.81 ± 1.47	0.0003
Fruits	−4.85 ± 1.89	−4.92 ± 1.83	0.1210
Dairy	−5.79 ± 0.84	−5.67 ± 1.03	0.0002
Soybean	−2.28 ± 2.87	−2.38 ± 2.89	0.2059
Meat	0.94 ± 2.89	0.17 ± 3.01	<0.0001
Fish	−2.32 ± 1.70	−2.53 ± 1.66	<0.0001
Egg	−1.46 ± 2.52	−1.24 ± 2.60	0.0007
Oil	2.33 ± 2.35	2.34 ± 2.38	0.8891
Salt	1.56 ± 1.83	1.86 ± 1.97	<0.0001
Alcohol	0.36 ± 1.36	0.40 ± 1.43	0.2843
Diet variety	−5.91 ± 1.63	−6.08 ± 1.76	<0.0001

### The risk of prediabetes in subjects with unfavorable dietary quality in every subgroup

**Figure 1:**
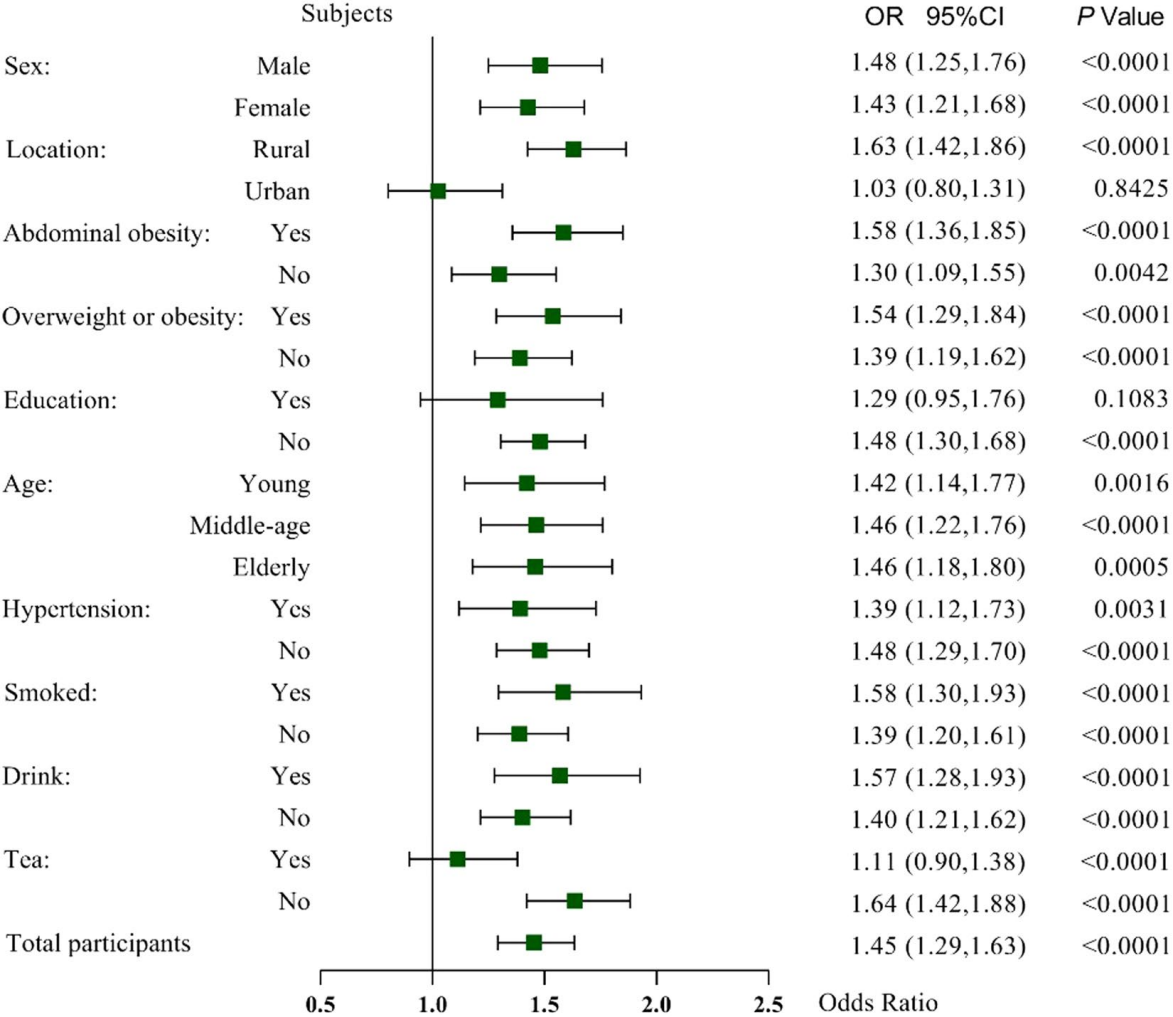
The risk of prediabetes associated with unfavourable dietary quality in the subgroup analysis.

Figure 1 shows that subjects with an unfavourable dietary quality had a higher risk of developing prediabetes (OR: 1.45, 95%CI: 1.29–1.63) after adjusting for energy, activity and all the variables shown in the figure. In the majority of subgroup analyses, subjects who had an unfavourable dietary quality had a higher risk of developing prediabetes, especially among the subjects who lived in rural areas (OR: 1.63, 95%CI: 1.25–1.76), those who had abdominal obesity (OR: 1.58, 95%CI: 1.36–1.85), those who were overweight or obese (OR: 1.54, 95%CI: 1.29–1.84), those who smoked (OR: 1.58, 95%CI: 1.30–1.93), those who consumed alcohol (OR: 1.57, 95%CI: 1.28–1.93) and those who did not drink tea (OR: 1.64, 95%CI: 1.42–1.88). Moreover, people with hypertension and older age also had a high risk of prediabetes.

## Discussion

The purpose of this cross-sectional study was to evaluate the dietary quality of people with prediabetes enrolled in the CHNS in 2009. We observed that diet imbalance was common among the participants, especially among people with prediabetes. Significant differences were found between the two groups in the HBS, LBS, and DQD scores; the intake of cereals, vegetables, dairy, meat, fish, egg, and salt; and diet variety. Moreover, people with prediabetes had moderate or high levels of dietary imbalance. Subjects with an unfavourable dietary quality had a higher risk of prediabetes in the majority of subgroups.

Over the past several decades, Chinese dietary patterns have undergone an ongoing transition. During this period, the intake of animal food, especially meat, has increased, along with a reduced intake of cereal^15^. Compared with previous studies^16^, people with prediabetes in our study had worse dietary quality, especially in regard to the excessive intake of food. Our results indicated that most participants in the two groups had an excessive intake of cereals, meat, oil and salt. Cereals remain a major staple food in China^17^, despite exhibiting a downward trend^15^. In our results, people with prediabetes had a higher mean percentage of consumption of cereals than people without prediabetes. Cereals are rich in carbohydrates that have a high glycaemic index (GI), and persons who consume a diet with an excessively high GI have a 30–40% increased risk of diabetes^18^. Meanwhile, Subjects without prediabetes had a greater intake of meat, and the main type of meat may be white meat. A systematic review reported that white meat could reduce the risk of cardiovascular disease, which is significantly associated with diabetes mellitus^19,20^. Moreover, the excessive consumption of processed red meat is associated with a higher incidence of diabetes and undetected diabetes mellitus^21,22^. People with prediabetes had more severely excessive intake levels of oil and salt. Excessive intake of oil could provide surplus energy, which may result in obesity^23^, and energy restriction seems to be the key for improving insulin action in type 2 diabetes patients^24^. Moreover, excessive intake of salt could lead to hypertension, which is associated with insulin resistance^25^.

In addition, our study showed that many participants had moderate or severe deficits in the intake of vegetables, fruits, dairy, fish, and eggs and diet variety. Although the consumption of these foods has been reported to be increasing during the last decade in China^17^, other studies indicated that the levels of consumption were still low^11^. In our study, the intake of vegetables, dairy, fish, and eggs and diet variety were significantly different between the two groups. The present study indicated that people with low levels of consumption of vegetables and fruits were more likely to develop prediabetes^26^. Additionally, the consumption of abundant vegetables and fruits could improve the survival of individuals with cancer^27^. Dairy, fish and eggs are good sources of high-quality protein, minerals, vitamins and fats. However, at the same time, we must pay attention to the excessive intake of cholesterol and saturated fatty acids, which were reported to be risk factors for cardiovascular disease and insulin resistance^28^.

Our results showed that people with an unfavourable dietary quality had a significantly increased risk of prediabetes in several subgroups, such as those stratified by sex, abdominal obesity, overweight or obesity, age, hypertension, smoking, drinking alcohol and drinking tea. Previous evidence showed that a balanced diet plus physical activity could reduce or delay the development of new type 2 diabetes^29^. In the subgroup of individuals from urban regions with high levels of education who drank tea, we did not observe the effect of unfavourable dietary quality on the risk of prediabetes. Previous studies showed that people who live in urban areas and have high education levels always have superior dietary quality than others^11^. These individuals can obtain food more conveniently, pay greater attention to nutritional information and have better cooking skills. Moreover, people who live in rural areas and who have low levels of education may lack nutrition knowledge and may also have difficulty obtaining abundant food. Previous studies have revealed that the consumption of tea is inversely associated with the risk of diabetes mellitus and its complications. Experimental studies have shown that tea has protective effects against diabetes by enhancing insulin action, ameliorating insulin resistance, scavenging free radicals, and decreasing inflammation^30^. Our results indicated that people who were obese (BMI) and had an unfavourable dietary quality had a high risk of prediabetes, and abdominal obesity and obesity (BMI) are significantly associated with an increased risk of diabetes, as they can cause abnormal glucose metabolism^31^. Subjects who smoked and drank alcohol had a high risk of prediabetes when they had an unfavourable dietary quality. A previous study showed that smoking and alcohol consumption were strongly correlated with prediabetes^32^. Therefore, to prevent the occurrence and development of prediabetes, we need to consume a balanced diet, change bad habits and avoid overweight and obesity.

In summary, a high HBS score implies the excessive intake of food, which often leads to multiple chronic diseases, especially hyperglycaemia. Meanwhile, a high LBS score indicates an insufficient intake of food, which causes nutritional deficiencies and ultimately results in dysglycaemia. Overall, a high DQD score implies poor food quality, which is associated with prediabetes. The DBI-16 could help people with prediabetes comprehend their dietary imbalance and guide medical departments in establishing prevention and treatment measures quickly and correctly for people at risk for prediabetes.

The significance of this study was that it was the first study to use the DBI scores to evaluate the dietary quality of Chinese people with prediabetes. We found that an unfavourable dietary quality was significantly associated with an increased risk of prediabetes. Limitations of this study also exist. First, we may have underestimated the intake of oil and salt. In our study, we calculated the individual intake of oil and salt based on consumption at home; this calculation excluded the intake of oil and salt outside the home. Second, the self-reported food intake of subjects may be inaccurate. Third, the data were collected in 2009 only, which naturally restricts the causal inference between dietary quality and prediabetes. Thus, we will conduct an additional study after the most recent data are released in the database. Fourth, the DBI-16 cannot be used to evaluate more detailed food categories, such as whole grains versus refined grains, which may have different effects on prediabetes.

## Conclusions

In conclusion, an imbalanced diet was common among the study population in China. The imbalances include the excessive intake of cereals, meat, oil and salt; the insufficient consumption of vegetables, fruits, dairy, fish, and eggs; and inadequate diet variety. Diet quality was significantly associated with an increased risk of prediabetes.

## Data Availability

The datasets analysed during the current study are available in the CHNS repository (https://www.cpc.unc.edu/projects/china/).
